# O & H’s Teeth

**Published:** 1858-04

**Authors:** 


### OLIVER & HARRISON'S PATENT TEETH.

Patented, May 20th, 1856, now free to the Profession. These are admirably adapted for the casting process, whether for cast plates, or swaged plates. We keep only the gum teeth, price 20 cents each.

Oliver's pamphlet of instructions, for mounting teeth by casting, price $1,00.

